# Glaucoma Characteristics and Influencing Factors during the COVID-19 Pandemic in the Huizhou Region

**DOI:** 10.1155/2023/8889754

**Published:** 2023-10-26

**Authors:** Huilan Zhou, Rui Liao, Dongxuan Zhang, Wei Wang, Shuifeng Deng

**Affiliations:** ^1^Huizhou Hospital Affiliated to Guangzhou Medical University (Huizhou Third People's Hospital), Huizhou 516000, Guangdong, China; ^2^State Key Laboratory of Ophthalmology, Zhongshan Ophthalmic Center, Sun Yat-sen University, Guangzhou 510000, Guangdong, China

## Abstract

**Objective:**

Glaucoma in individuals who tested positive for the coronavirus disease 2019 (COVID-19) during the pandemic outbreak has not been comprehensively studied. Therefore, this study aimed to analyze the characteristics and risk factors of glaucoma during the COVID-19 pandemic in Huizhou.

**Methods:**

Retrospective data from outpatients with glaucoma at the Huizhou Hospital Affiliated with Guangzhou Medical University and Longmen County People's Hospital were collected during two periods: the COVID-19 pandemic period (Phase A: December 1, 2022, to January 19, 2023) and the prevention and control period (Phase B: December 1, 2021, to January 19, 2022). The demographic characteristics of the outpatients during both phases were compared. The characteristics of glaucoma in patients with COVID-19 during Phase A were examined. Multivariate logistic regression analysis was used to identify factors influencing the development of acute angle-closure glaucoma (AACG) in Phase A patients.

**Results:**

The proportion of patients with glaucoma was significantly higher during Phase A than during Phase B at both hospitals. No statistically significant differences were observed between patients with glaucoma during Phases A and B for age, sex, and region. A high COVID-19-positive rate was associated with old age, females, AACG, newly diagnosed glaucoma, and binocular involvement during phase A. Females testing positive for COVID-19, glaucoma that started after testing positive for COVID-19, and a history of medication use were associated with a higher proportion of AACG in phase A. Multivariable logistic regression analysis identified testing positive for COVID-19 as an independent potential risk factor for developing AACG.

**Conclusion:**

In summary, during the COVID-19 pandemic in Huizhou, patients with COVID-19 were primarily affected by AACG, especially females, older individuals, and those with binocular involvement. Testing positive for COVID-19 increases the risk of developing AACG.

## 1. Introduction

The coronavirus disease 2019 (COVID-19) pandemic has significantly affected the lives and health status of the Chinese population. COVID-19 originated in Wuhan, China, in December 2019 and was declared a global pandemic by the World Health Organization in March 2020 [1]. While other countries experienced the peak of the COVID-19 pandemic in 2021, China implemented measures to prevent the resurgence, both locally and internationally, from December 2019 to December 2022. However, as domestic policies were gradually relaxed by December 2022, the outbreak continued to surge in different parts of China, including the Huizhou region of Guangdong Province. A major outbreak occurred between December 1, 2022, and January 19, 2023. Consequently, healthcare-seeking behaviors, daily habits, and mental states of people have changed. Specifically, people began to have prolonged indoor activities, reduced outdoor activities, increased use of preventive over-the-counter cold medications, increased anxiety and tension, and were reluctant to seek medical attention unless necessary.

After the COVID-19 outbreak, significant changes in outpatient visits and treatment plans were observed. Notable changes were observed in the incidence, epidemiological characteristics, and proportion of patients with glaucoma. In countries such as the United States and India, a noticeable increase in the number of patients with acute angle-closure glaucoma (AACG) during the COVID-19 outbreak was observed [2]. Moreover, changes were observed in the methods used to treat glaucoma [3]. Several factors may have contributed to this, including policy changes, increased self-awareness, lifestyle changes among individuals with COVID-19, and medication-related factors that can lead to the onset of acute glaucoma [4, 5]. AACG is considerably influenced by external and psychological factors and is characterized by sudden onset and severe functional damage. Moreover, AACG has received significant attention as a comorbidity during the COVID-19 pandemic.

Experts have argued that a general delay in providing treatment to patients with acute glaucoma has existed since the beginning of the COVID-19 pandemic [6]. This delay can lead to permanent vision loss in 72% of patients [6]. Therefore, it is important to have a comprehensive understanding of the characteristics of glaucoma, particularly AACG, during the outbreak and identify the factors that contribute to its occurrence. This understanding will allow a comprehensive investigation of the risk factors of glaucoma and treatment strategies. However, previous studies, both locally and internationally, have primarily focused on analyzing the challenges faced by patients with glaucoma in terms of follow-up care during the COVID-19 pandemic, changes in surgical approaches, and treatment modifications [3, 7–9]. Limited research has examined the characteristics and potential factors influencing the development of glaucoma, specifically AACG, in patients who tested positive for COVID-19 during the outbreak. A comprehensive understanding of the specific traits of glaucoma and various influencing factors could be valuable in developing innovative prediction approaches to address potential challenges in preventing glaucoma in future waves of the pandemic. Therefore, this study aimed to analyze the characteristics of glaucoma and its related influencing factors during the COVID-19 pandemic in Huizhou City, a well-developed city in the Pearl River Delta region of Guangdong Province, China.

## 2. Materials and Methods

### 2.1. Study Design and Population

This retrospective observational study was performed in accordance with the guidelines of the Declaration of Helsinki and was approved by the hospital's Ethics Committee (2023-KY-004). Data were collected from the outpatient electronic information systems of two hospitals: Longmen County People's Hospital (Hospital 1), situated in the county area, and Huizhou Hospital Affiliated with Guangzhou Medical University (Hospital 2), located in the city center. The study was divided into two periods: the COVID-19 outbreak (phase A; from December 1, 2022, to January 19, 2023) and prevention and control (phase B; from December 1, 2021, to January 19, 2022) periods. Per the regional policy for epidemic prevention and control, all patients (phases A and B) visiting the hospital were required to provide and register their COVID-19 nucleic acid or antigen test results (positive or negative) in the outpatient electronic information system.

**Figure 1:**
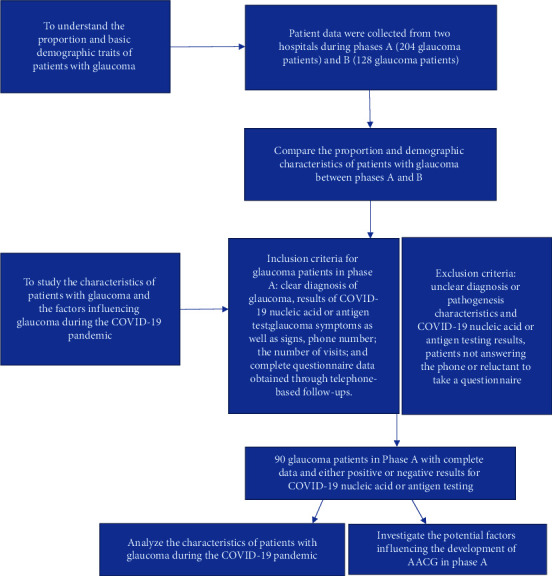
Flow diagram of the study participants.

We enrolled patients with glaucoma who visited the ophthalmology outpatient department. To understand the proportion and basic demographic traits of patients with glaucoma between phases A and B, inclusion criteria included diagnosis of glaucoma, sex, age, and region, which were collected from outpatient electronic records. To understand factors influencing glaucoma during the COVID-19 pandemic, the patients in phase A had additional inclusion criteria including information collected on specific subtypes of glaucoma, such as AACG, chronic angle-closure glaucoma, and open-angle glaucoma. Phase A patients also required documentation of glaucoma symptoms, such as eye pain, blurred vision, headache, nausea, and vomiting, and signs on slit-lamp examination, such as corneal edema, anterior chamber depth, iris and pupil status, visual acuity, and intraocular pressure. Participant phone number, the number of previous visits, and whether the patient was newly diagnosed were recorded, and complete questionnaire data were obtained through telephone-based follow-ups. The study description is summarized in Figure 1.

Patients were excluded from the study based on the following criteria: the diagnosis or pathogenesis characteristics were not clearly documented in the outpatient electronic information system; COVID-19 nucleic acid or antigen testing results were unclear; and patients did not answer the phone or refused to take a questionnaire.

AACG was defined as the presence of at least one typical symptom of primary AACG, including pain, blurred vision, seeing halos, nausea, and vomiting. Additionally, the following signs on slit-lamp examination were required: corneal edema, shallow anterior chamber depth, unreactive mid-dilated pupil, and intraocular pressure greater >21 mmHg [10]. Nonacute angle-closure glaucoma (N-AACG) was recorded in comparison to patients with AACG.

### 2.2. Data Collection

Data from patients with glaucoma were collected using outpatient electronic information systems and follow-up calls. To determine whether there were any changes in the proportion and basic demographic traits of patients with glaucoma between phases A and B, we collected information on the number of patients with glaucoma and the total number of outpatients who visited the two hospitals, as well as data on sex, age, and region.

Another objective was to study the characteristics of patients with glaucoma and the factors influencing glaucoma during the COVID-19 pandemic. To achieve this, we collected data on patients with glaucoma: In phase A, we gathered information on whether glaucoma affected one or both eyes, whether the onset of glaucoma was sudden or gradual, whether glaucoma was newly diagnosed, and the patients' COVID-19 status (positive or negative). We also conducted a questionnaire-based survey that included questions about whether glaucoma started after testing positive for COVID-19, if there was any activity in a dark room, poor appetite, or being in the prone position before the onset of glaucoma. The questionnaire additionally assessed if the patients used any medications after testing positive for COVID-19, and if the patients had any non-COVID-19-related systemic diseases. In phase B, owing to policy restrictions, we did not find any patients with glaucoma who tested positive for COVID-19. Therefore, the survey mainly focused on the patients in Phase A. Multiple visits by the same patient were consolidated into a single recording. Patients who were newly diagnosed with glaucoma without any prior history of the condition were categorized as having newly diagnosed glaucoma. The flowchart illustrates our analysis and approach (Figure 1).

In this study, we classified regions based on whether the patients' place of residence was within or outside a 20-km radius from the hospital [8]. Adverse behavior was defined as patterns of COVID-19 infection-related behavior in a darkroom for more than 1 hour, having a poor appetite, and being in the prone position [11–13]. History of medication use was defined as the use of traditional Chinese medicine, cough suppressants, or a combination of both [14]. NonCOVID-19-related systemic diseases included hypertension, diabetes, rheumatism, and other related conditions [15].

### 2.3. Statistical Analysis

Statistical analyses were performed using IBM SPSS (version 22.0; IBM Corp., Armonk, N.Y., USA) and SAS (version 9.4; SAS Inc., Cary, N.C., USA) software. Independent-sample Pearson Chi-square tests were used to compare the proportion of patients with glaucoma in the ophthalmology department during the two phases. The proportion rate difference (RD) was computed, and the 95% confidence interval (CI) of RD was estimated using the Newcombe and Robert G method [16]. Differences in demographic characteristics between Phases A and B were determined using independent-sample *t*-tests for continuous variables and chi-square tests for categorical variables. The same methods were used to examine the differences in the characteristics of glaucoma between patients who tested positive and negative for COVID-19 during phase A. To investigate the factors influencing the development of AACG, a comprehensive analysis was conducted. First, we used *t*-tests for continuous variables and chi-square tests for categorical variables to compare the differences in these potential factors between the AACG and non-AACG groups. Second, binary logistic regression was used to identify potential factors, and crude odds ratios (ORs) and 95% CIs were calculated. Third, variables with *P* < 0.05 in the univariate model were selected as candidate influencing factors to construct a multivariate model to estimate the adjusted OR and 95% CI. In the case of a small sample size and when some of the cells formed by the outcome and categorical predictor variables had no observations, the exact logistic regression method was applied to estimate the parameter, which was based on appropriate permutational distributions of sufficient statistics [17]. The significance level for the above analysis tests was set at *P* < 0.05.

## 3. Results

This study found a significant difference in the proportion of patients with glaucoma during phases A and B in hospitals 1 and 2. Specifically, a statistically significant increase in the proportion of patients with glaucoma was observed during Phase A than during Phase B (RD [95% CI] = 2.22% [1.95–2.92%]; *P* < 0.001) (Table 1). Furthermore, no statistically significant differences were found in age, sex, or region between patients with glaucoma during Phases A and B (all *P* > 0.05) (Table 2).

Regarding COVID-19 test status, statistically significant differences were observed in age, sex, monocular/binocular involvement, AACG/N-AACG, and newly diagnosed glaucoma between patients who tested positive and negative for COVID-19 during Phase A. Particularly, patients with glaucoma who tested positive for COVID-19 were older than those who tested negative. Moreover, the COVID-19-positive rate was higher among female patients and patients with AACG, newly diagnosed glaucoma, and binocular involvement during Phase A (Table 3).

In additio, 90 patients with glaucoma during Phase A were divided into the AACG and N-AACG groups, and the potential risk factors influencing the development of AACG in Phase A were analyzed. The results showed statistically significant differences between the two groups in terms of sex, COVID-19 status, whether glaucoma started after testing positive for COVID-19, and history of medication use. Specifically, in female patients with COVID-19, glaucoma started after testing positive for COVID-19, and patients with a history of medication use had a higher proportion of AACG cases (Table 4).

Finally, multivariate logistic regression models were used to analyze factors affecting AACG development. The results showed that the COVID-19 status was an independent risk factor for the development of AACG when adjusted for other confounders (OR (95% CI) = 0.089 (0.002–0.845); *P*=0.030) (Table 5).

## 4. Discussion

This study examined the characteristics of glaucoma in patients who tested positive for COVID-19 and investigated factors that increased the risk of AACG in these patients during a specific period of the pandemic. This study suggests that COVID-19 could potentially contribute to the development of AACG. This study is the first to describe the characteristics of glaucoma and the potential risk factors for developing AACG in the Huizhou region of Guangdong Province during the COVID-19 period. Although the mechanism underlying COVID-19-induced AACG has not been clarified, this study aims to create awareness of preventive measures against acute glaucoma.

Since the beginning of 2020, the world has faced increasing difficulties because of the COVID-19 pandemic. Many governments implemented national or local lockdown measures to curb the spread of the causative virus and reduce the strain on hospitals. In addition, strict home isolation was advised for “clinically vulnerable” individuals, including those aged ≥70 years, and “clinically extremely vulnerable” individuals receiving immunosuppressive therapy [18, 19]. Moreover, factors such as the COVID-19 outbreak, travel restrictions, and economic hardships may have influenced patients' healthcare-seeking behaviors [2, 20]. Poyser et al. reported a 53% decline in visits to the ophthalmic emergency department during the pandemic, whereas a study conducted in India revealed a significant decrease of 78.9% in overall visits to ophthalmic outpatient clinics, along with a noteworthy increase of 62.4% in glaucoma-related emergency visits [2, 21]. The decrease in overall outpatient visits during the COVID-19 outbreak can be attributed to multiple factors, such as government policies and heightened awareness of the risks of infection [4, 5]. However, patients with glaucoma may be more motivated to seek immediate medical attention because of the specific symptoms they experience, such as severe eye pain, headaches, and visual impairment [6]. Our study found a significant reduction in the total number of outpatient visits during the COVID-19 pandemic, which is consistent with previous research [2, 6]. However, a substantial increase was observed in the proportion of patients with glaucoma attending outpatient departments in the Huizhou region after the pandemic. This highlights the potential effect of the COVID-19 pandemic on the prevalence of glaucoma and emphasizes the importance of considering the temporal relationship between the pandemic and glaucoma within the region.

Demographic factors remained consistent in both periods, as no significant differences were observed in terms of age, sex, and region between Phases A and B. This suggests that the distribution of glaucoma cases did not vary significantly based on these variables. Further analysis of patients with glaucoma in Phase A revealed that testing positive for COVID-19 was primarily associated with AACG development, females, older age, binocular status, and newly diagnosed cases. Studies from other countries have also reported a higher prevalence of AACG in elderly females, which aligns with our findings [22, 23]. However, previous studies have rarely reported cases of sudden attacks in either eye, which are typically associated with systemic diseases or drug use [15, 24–26]. Notably, patients with glaucoma who tested positive for COVID-19 in this study presented with simultaneous binocular involvement, indicating a higher risk of vision loss [11]. This finding may also explain why patients with glaucoma who tested positive for COVID-19 in this study were primarily newly diagnosed rather than previously diagnosed patients, as they experienced acute-onset glaucoma affecting both eyes, which poses a significant threat to their vision and quality of life [11]. AACG is a specific type of angle-closure glaucoma characterized by the abrupt closure of the chamber angle, leading to a sudden rise in intraocular pressure, which is accompanied by intense eye pain, a severe headache, and significant visual impairment [10, 27, 28]. Patients with these symptoms require immediate medical care.

Moreover, our study suggests that AACG is associated with COVID-19-positive status, and the onset of AACG often occurs after a positive diagnosis of COVID-19, suggesting a potential relationship between COVID-19 and the development of AACG. Further multivariate analysis suggested that COVID-19-positive status was an independent potential risk factor for developing AACG. However, how COVID-19 positivity induces AACG remains unclear. Our hypothesis is based on the results of previous studies [10, 15], which suggest that AACG can be triggered by various factors, including drug use and systemic disease [10, 15, 26]. In our study, univariate analysis showed no association between AACG onset and systemic diseases, such as hypertension, diabetes, and rheumatism. However, there was an association between AACG onset and a history of medication use. Although history of medication use was not an independent risk factor in the multivariate regression analysis, we believe that this may be because history of medication use is indirectly correlated with AACG [15], as most patients use drugs after testing positive for COVID-19. After testing positive for COVID-19, many patients rely on traditional Chinese medicine or cough suppressants to alleviate symptoms, including fever and cough [14, 29]. Other analyses have shown that certain ingredients in traditional Chinese medicine contain ephedrine-like drugs that can cause AACG through ciliary edema [30]. Similarly, cough suppressants contain anticholinergic components that can lead to mydriasis and angle closure, resulting in AACG [10]. Although our study did not identify the specific drugs that can induce AACG, results suggest that post-COVID-19 medications may potentially trigger the development of severe glaucoma. Furthermore, previous studies have suggested that COVID-19 infection-related adverse behavior patterns, such as spending more time in dark rooms, lying in a prone position, and having a poor appetite leading to hyponatremia, can cause acute primary angle-closure glaucoma in both eyes [11–13]. However, these reports were based on hospitalizations for severe COVID-19 infection, and the outpatients with glaucoma in our study rarely exhibited such typical behavioral patterns. Therefore, COVID-19-related behavior patterns may not be the primary factor contributing to AACG development among outpatients during the pandemic. Moreover, COVID-19 may induce AACG through alternative mechanisms.

Considering the strong association between testing positive for COVID-19 and developing AACG, we hypothesized that COVID-19 may affect the function of the angle, leading to glaucoma onset. Previous studies have indicated that viral infections can trigger AACG [31, 32]. Infections such as Vogt-Koyanagi-Harada syndrome and human immunodeficiency virus infection are more likely to induce AACG, and Hantavirus infection specifically causes acute binocular angle-closure glaucoma, possibly mediated by choroidal leakage [31, 32]. Studies have also found that acute glaucoma may be an initial sign of acute retinal necrosis, requiring high-dose antiviral treatment to prevent disease progression [28]. Secondary inflammation and viral iridocyclitis may contribute to the development of acute glaucoma. There is evidence that the severe acute respiratory syndrome coronavirus-2 (SARS-CoV-2) infection has been detected in the corneas of deceased COVID-19 patients [33]. However, the sensitivity of SARS-CoV-2 detection in the eye tissue and ocular fluid is relatively low [34]. Therefore, based on these theories, we speculated that COVID-19 may affect the function of the angle to a certain extent, leading to glaucoma; however, additional cases and advanced detection methods are needed to explore the specific mechanisms of COVID-19 in glaucoma.

Our study had some limitations. First, our analysis was retrospective and descriptive, which means that we were unable to account for certain factors that may influence the onset of glaucoma, particularly those related to eye anatomy. The study was limited by the lack of standardized methods to consider all known risk factors for acute glaucoma. Second, the data used in our study were obtained from a specific geographical location (Huizhou), with a relatively high rate of loss during telephone-based follow-ups, which may not fully represent ophthalmic cases in other cities or provinces. Consequently, the generalizability of our findings to other regions may be limited. Future studies should include data from other regions to enhance the validity of these findings. Finally, large-scale, multicenter, controlled studies are needed to thoroughly compare the characteristics of acute glaucoma in infected and uninfected patients with COVID-19 to provide a more comprehensive understanding of the topic.

## 5. Conclusions

This study identified a relationship between testing positive for COVID-19 and the development of glaucoma. The results emphasize the need to acknowledge and properly address eye-related complications that can arise from COVID-19. This understanding could aid in the development of new approaches to anticipate and prevent the incidence of glaucoma in future waves of the pandemic.

## Figures and Tables

**Table 1:** Proportion of patients with glaucoma in the ophthalmology department of two hospitals during phases A and B.

Study site	Phase A, *n* (%)^b^	Phase B, *n* (%)^b^	RD (95% CI)^a^ (%)	*χ* ^2^	*P*
Hospital 1 (*N* = 2937)	68 (5.01)	43 (2.72)	2.26 (0.86–3.67)	10.474	0.001
Hospital 2 (*N* = 7877)	136 (4.04)	85 (1.88)	2.16 (1.38–2.93)	32.909	<0.0001
Combined (*N* = 10814)	204 (4.32)	128 (2.10)	2.22 (1.95–2.92)	43.971	<0.001

*Note.* Proportion of patients with glaucoma (%) = number of patients with glaucoma/total number of outpatient visits to different study site of different phase × 100%. ^a^RD = rate difference, 95% CI = 95% confidence interval. The 95% CI of RD was estimated using the Newcombe and Robert G method [16]. ^b^, *n*(%) = the number and proportion of patients with glaucoma. Pearson's chi-square test was used to determine the proportion of patients with glaucoma between phases A and B.

**Table 2:** Demographic characteristics of patients with glaucoma during phases A and B.

Demographic characteristics	Phase A (*N* = 204)	Phase B (*N* = 128)	*χ* ^2^/*t*	*P*
Age, years (mean ± SD)	58.21 ± 15.54	59.05 ± 16.68	−0.470	0.640^a^
Sex, *n* (%)			1.353	0.245^b^
Male	95 (58.28)	68 (41.72)		
Female	109 (64.50)	60 (35.50)		
Region, *n* (%)			0.001	0.972^b^
Distance from the patient's home within 20 km	104 (61.54)	65 (38.46)		
Distance from the patient's home outside 20 km	100 (61.35)	63 (38.65)		

*N*: number of patients with glaucoma; SD: standard deviation. *n* (%) = number and percentages of patients with glaucoma for each categorical group. Independent-sample *t*-test (^a^) and Pearson's chi-square test (^b^) were used to test the differences in demographic characteristics of patients with glaucoma between phases A and B.

**Table 3:** Characteristics of patients with glaucoma in the COVID-19-positive and COVID-19-negative groups during phase A.

Characteristics	COVID-19 positive (*N* = 49)	COVID-19 negative (*N* = 41)	*χ* ^2^/*t*	*P*
Age, years (mean ± SD)	64.29 ± 13.34	55.49 ± 17.15	−2.737	0.007^a^
Sex, *n* (%)			4.997	0.025^b^
Male	16 (41.03)	23 (58.97)		
Female	33 (64.71)	18 (35.29)		
Region, *n* (%)			0.004	0.951^b^
Distance from the patient's home within 20 km	29 (54.72)	24 (45.28)		
Distance from the patient's home outside 20 km	20 (54.05)	17 (45.95)		
Monocular/binocular involvement, *n* (%)			4.897	0.027^b^
Monocular	15 (40.54)	22 (59.46)		
Binocular	34 (64.15)	19 (35.85)		
AACG/N-AACG involvement, *n* (%)			16.295	<0.001^b^
AACG	20 (95.24)	1 (4.76)		
N-AACG	29 (42.03)	40 (57.97)		
Newly diagnosed glaucoma, *n* (%)			6.473	0.011^b^
Yes	22 (73.33)	8 (26.67)		
No	27 (45.00)	33 (55.00)		

*Note. n* (%), number of patients with glaucoma; %, ratio of the patients. SD = standard deviation. The independent-sample *t*-test (^a^) and Pearson's chi-square test (^b^) were used to test the difference between COVID-19 positive and COVID-19 negative groups. AACG, acute angle-closure glaucoma; N-AACG, nonacute angle-closure glaucoma.

**Table 4:** Potential risk factors for the development of AACG in the AACG group compared with the N-AACG group during phase A.

Potential risk factors	AACG group (*N* = 21)	N-AACG group (*N* = 69)	*χ* ^2^/*t*	*P*
Sex, *n* (%)			6.579	0.010^b^
Female	17 (33.33)	34 (66.67)		
Male	4 (10.26)	35 (89.74)		
Age, years (mean ± SD)	65.86 ± 8.63	58.58 ± 17.01	−1.883	0.063^a^
COVID-19 status, *n* (%)			18.378	<0.001^c^
Negative	1 (2.44)	40 (97.56)		
Positive	20 (40.82)	29 (59.18)		
Glaucoma started after testing positive for COVID-19, *n* (%)			20.649	<0.001^b^
No	7 (10.77)	58 (89.23)		
Yes	14 (56.00)	11 (44.00)		
Adverse behavior, *n* (%)			—	0.233^c^
No	20 (22.47)	69 (77.53)		
Yes	1 (100.00)	0(0.0)		
History of medication use, *n* (%)			6.988	0.008^b^
No	9 (15.00)	51 (85.00)		
Yes	12 (40.00)	18 (60.00)		
Monocular/binocular involvement, *n* (%)			0.034	0.853^b^
Monocular	9 (24.32)	28 (75.68)		
Binocular	12 (22.64)	41 (77.36)		
Non-COVID-19 systemic diseases, *n* (%)			1.860	0.173^b^
No	17 (27.42)	45 (72.58)		
Yes	4 (14.29)	24 (85.71)		

*Note. n* (%), Number of patients with glaucoma; %: ratio of the patients. SD = standard deviation. An independent-sample *t*-test (^a^) for age, Pearson's chi-square test (^b^), and Fisher's exact test (^c^) for categorical variables were used to test the difference between the AACG and N-AACG groups. AACG, acute angle-closure glaucoma; N-AACG, nonacute angle-closure glaucoma.

**Table 5:** Univariate and multivariate logistic regression model of the potential factors influencing the development of AACG during phase A.

Potential risk factors	Univariate model		Multivariate model^a^	
	Crude OR (95% CI)	*P*	Adjusted OR (95% CI)	*P*
Sex (ref: male)	4.308 (1.234–19.414)	0.018	2.609 (0.595–13.817)	0.257
Age	1.036 (0.999–1.079)	0.056		
COVID-19 status (ref: positive)	0.037 (<0.001–0.260)	<0.001	0.089 (0.002–0.845)	0.030
Glaucoma started after testing positive for COVID-19 (ref: no)	10.168 (3.051–37.587)	<0.001	3.136 (0.786–13.809)	0.119
Adverse behavior (ref: no)	3.286 (0.173-inf)^*∗*^	0.467		
History of medication use (ref: no)	3.715 (1.210–11.888)	0.019	1.024 (0.235–4.132)	>0.999
Monocular/binocular involvement (ref: monocular)	0.912 (0.305–2.805)	>0.999		
NonCOVID-19 systemic diseases (ref: no)	0.445 (0.098–1.580)	0.272		

*Note.* OR = odd ratio, 95% CI = 95% confidence interval. In the univariate model, only one candidate variable was entered into the logistic regression analysis, and the crude OR (95% CI) was calculated. ^*∗*^Because the data were sparse, the lower limit could not be calculated. (^a^) Variables with *P* < 0.05 in the univariate model were selected as candidate influencing factors to construct a multivariate model to estimate the adjusted OR and 95% CI. Model evaluation: Hosmer–Lemeshow goodness-of-fit test, *χ*^2^ = 5.509, *P*=0.357, which indicated a good model fit. AACG, acute angle-closure glaucoma; N-AACG, nonacute angle-closure glaucoma.

## Data Availability

Data used to support the findings of this study are available from the corresponding author upon request.
